# Vertical Excitation Energies and Lifetimes of the Two Lowest Singlet Excited States of Cytosine, 5-Aza-cytosine, and the Triazine Family: Quantum Mechanics–Molecular Mechanics Studies

**DOI:** 10.1021/acs.jctc.2c01262

**Published:** 2023-03-24

**Authors:** Ondřej Tichý, Marek Pederzoli, Jiří Pittner, Jaroslav V. Burda

**Affiliations:** † Department of Chemical Physics and Optics, Faculty of Mathematics and Physics, Charles University, Ke Karlovu 3, 121 16 Prague 2, Czech Republic; ‡ J. Heyrovský Institute of Physical Chemistry, Academy of Sciences, Dolejškova 3, 182 23 Prague 8, Czech Republic

## Abstract

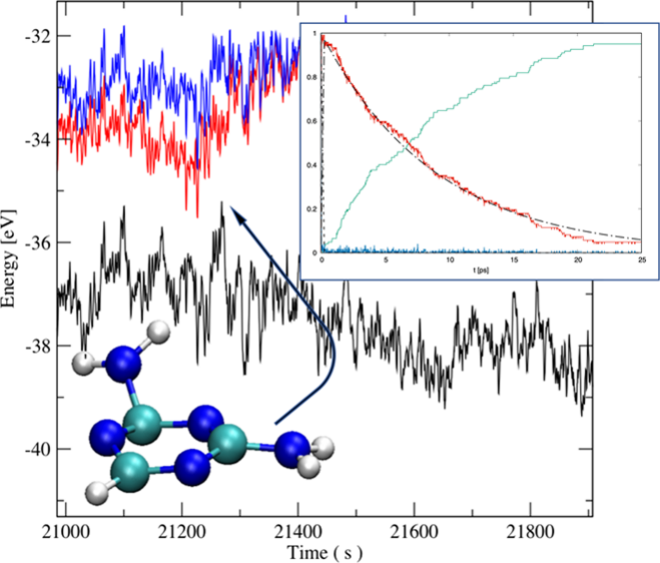

A swarm of semi-classical quantum mechanics/molecular mechanics molecular-dynamics simulations where OM2/MNDO is combined with the Gromacs program for consideration of explicit water is performed, solving the time-dependent Schrödinger equation in each step of the trajectories together with the Tully’s fewest switches algorithm. Within this stochastic treatment, time dependent probabilities of the three lowest electronic states are determined. The fact that nucleobases are quickly deactivated is confirmed in the cytosine case where our best lifetime estimation is τ_1_=0.82 ps for the model with 100 water molecules with the SPCE force field and a time step of 0.1 fs. Lifetimes of the remaining molecules are visibly longer: 5-azacytosine, 2,4-diamino-1,3,5-triazine (DT), and 2,4,6-triamino-1,3,5-triazine (TT) molecules have an S_1_ → S_0_ de-excitation time of slightly above 10 ps. The lifetimes of the triazine family increases with the increasing number of exocyclic amino groups, that is, *s*-triazine < 2-amino-1,3,5-triazine < DT < TT. This can be explained by a higher mobility of the carbon-bonded hydrogen atoms in comparison with heavier amino groups since their movement is slowed down due to a substantially higher mass than hydrogen atoms, which can easier reach the out-of-plane positions required in the conical intersection structures. Moreover, bulkier NH_2_ ligands suffer due to greater friction caused by the surrounding water environment. These mechanical aspects caused a change in the explored lifetime dependences in comparison with our previous gas-phase study.

## Introduction

Recently, a lot of work has been focused on time-resolved spectra of nucleic bases, which are known for their very short lifetimes of excited states.^1^ The aza-derivatives of cytosine and other related molecules (e.g., triazine derivatives) have substantially longer lifetimes measured by time-resolved spectroscopy.^2^ Lately, several other ultrafast experiments on similar topics have also been performed in the same laboratory.^3^ In order to explore photodynamic properties, time-resolved photoelectron spectroscopy (TRPES) represents one of the fundamental methods. It allows for examining dynamical behavior of molecules in excited states.^4^ Ulrich et al.^1^ examined all three pyrimidine nucleobases and showed that a very fast decay (<50 fs) of the S_2_ (π–π*) state to the S_1_ (*n*–π*) state can be observed. This process is followed by a substantially slower decay (820 fs for cytosine, 490 fs for thymine, and 530 fs for uracil); later, a few-picosecond channel (3.2 ps for cytosine, 6.4 ps for thymine, and 2.4 ps for uracil) was also found. By employing ultrafast transient absorption spectroscopy, Pecourt et al.^5^ explored the excited-state dynamics of DNA nucleosides. They found that pyrimidine bases have a significantly longer lifetime than purine bases: τ = 720 fs for cytidine and 540 fs for thymidine compared to τ = 290 fs for adenosine and 460 fs for guanosine. Nevertheless, the excited state dynamics of cytosine tautomers was studied by Kosma et al.,^6^ observing a de-excitation of cytosine of higher than 150 ps. They showed evidence that it is really cytosine, not its tautomers, that de-excites so late. However, there is still a possibility that some of the tautomers need time to recombine back to the keto form. Ma et al.^7^ report a similar long-time non-radiative decay of cytosine and its derivatives especially in methanol solution, which substantially prolongs the de-excitation (ca. 5 times) in comparison with a water solution. Ultrafast IR spectroscopy was applied on the excitation of cytosine derivatives, confirming conclusions of previous studies.^8^ The role of methylation by adding a methyl ligand to the 5-position in deoxycytidine in solution on the excitation lifetime was explored by Martínez-Fernández et al.^9^ whose experimental data was supported by CASPT2 and TD-CAM-B3LYP calculations. Melamine (triaminotriazine) femtosecond transient absorption spectroscopy was applied in determining the lifetime for S_1_ → S_0_ of ca. 13 ps.^10^ Zhang et al.^10^ found the lifetime of the S_1_ excited state of triaminotriazine to be 13.3 ps using time-resolved IR spectroscopy. Based on preliminary TD-DFT calculations, they concluded that the lowest-energy ^1^π–π* transition is forbidden while the transition to the ^1^*n*–π* state was found to have a significant oscillator strength—the absorption spectrum rises monotonically from 260 to 210 nm with a weak shoulder at 240 nm.

Concerning computational studies on cytosine, Merchán et al.^4,11^ studied individual electronic surfaces and their crossings at the CASPT2 computational level, including also singlet–triplet coupling. They showed that the potential energy surface (PES) of the lowest π–π* excitation does not cross with any other surface in the close vicinity of the optimal structure. However, slightly further from the Franck–Condon area, the excited state based on the *n*_O_–π* transition can be already found below the π–π* PES. A very extensive and systematic examination of cytosine was conducted by González and González-Vázquez.^12^ A very detailed analysis of various methods and basis sets for the determination of the vertical energies was combined with ab initio MD of the two lowest excited states, confirming very fast de-excitation dynamics.^12a^ In their next paper, a possibility of de-excitation by an intersystem crossing involving triplet states was explored.^12b^ The deactivation pathway through triplet states was found to be two times slower compared to the direct S_1_ → S_0_ process. Also, another possibility of decay via a simultaneous three-state S_2_/S_1_/S_0_ conical intersection is proposed in analogy with studies of Blancafort and Robb^13^ and Kistler and Matsika.^14^ The developed methodology was also applied to uracil,^15^ 2-thiouracil, and thymine.^16^ Ab initio simulations were lately used for investigation of 5-azacytosine^17^ where electronic transition energies of the two lowest singlet and triplet states were examined. It was found that the intersystem crossing influences the relaxation process to the ground state only marginally.

Since the modified six-membered ring molecules already represent a relatively large electronic system for accurate DFT or even ab initio approaches, the semi-empirical OM2/MNDO method can be chosen as a convenient tool for the determination of electronic structures especially in combination with a molecular mechanics description of the solvent environment. An important feature of such a model is the possibility to describe the ground and excited states with the MRCI/GUGA^18^ algorithm. The estimation of lifetimes of chosen molecules is determined by hopping dynamics simulations, starting the time evolution from the second excited state. To demonstrate the ability of the chosen method to correctly describe excited-state energies with sufficient accuracy, the vertical excitation energies of the optimized heterocycles were explored in a previous study and benchmarked against the TD-DFT and post-HF methods. In this way, the plausibility of the introduced computational model was clearly confirmed.

There are two main goals for our contribution: first, the development of a new computational tool in order to connect the semi-empirical MNDO2020 program and the Gromacs code for molecular dynamics. The new environment has been added to the Newton-X v 2.2 interface and will be publicly available to the computational community. The second objective is to explain the role of explicit solvent molecules. In our study, cytosine (Cyt), 5-azacytosine (5AC), and the triazine family, namely, 1,3,5-triazine also named as *s*-triazine (*s*T), 2-amino-1,3,5-triazine (AT), 2,4-diamino-1,3,5-triazine (DT), and 2,4,6-triamino-1,3,5-triazine (TT), are examined. To determine the lifetimes of the first two excited states of these molecules, a stochastical approach is chosen in simulating OM2/MNDO hopping dynamics, employing a swarm of more than 100 trajectories for each molecule. At this semi-empirical level, the non-adiabatic dynamics is evaluated. The probability of interstate hopping is checked in every MD step by solving the time-dependent Schrödinger equation combined with Tully’s fewest switches algorithm.^19^ In our previous study, these molecules were evaluated in the gas phase.^20^ Here, the QM/MM methodology is implemented where, in the QM part, the MRCISD electronic states of the explored molecules are determined using the MNDO2020 program. The solute molecule is considered under the influence of the solvent embedding, using explicit water molecules evaluated within the Gromacs framework by classical molecular dynamics. Cooperation between both codes is realized by the Newton-X interface as stated above.

## Computational Details

The lifetimes of the first two excited states were determined by the OM2^21^/MNDO^22^ semi-empirical method, applying the program MNDO2020 from Thiel laboratory.^23^ Within this method, multireference CISD calculations with an active space consisting of four highest occupied orbitals and three lowest unoccupied orbitals (labeled (8,7) according to modern notation) are used to evaluate the excited-state energies of the given molecule. The active space is chosen based on the MO analysis performed in the previous study^20a^ where at least two MOs with a π character in both virtual and occupied spaces are considered. This setting is applied for evaluating ground-state trajectories of the explored molecules in explicit solvent treated within the hybrid QM/MM approach at temperature *T* = 298 K. In the MM shell, 100 water molecules are considered, employing the SPCE model in the Gromacs program v 5.1.1.^24^ The combining interface is provided by Newton-X v 2.2,^25^ which adopts results from MNDO99 and MNDO2020 programs into the QM core. An Andersen thermostat^26^ is applied on the solvent molecules only. Forces acting on individual atoms are determined from (negatively taken) energy gradients, summing up contributions from both QM and MM parts. At first, the ground-state molecular dynamics (MD) is performed with a time step of 0.5 fs. Usually, 10 ps long trajectories were found to be sufficient for the phase-space search. Based on these trajectories, a swarm of structures is obtained for subsequent hopping dynamics starting from the second excited state. Such a swarm consists of over 100 hopping dynamics trajectories. There are two criteria applied for a selection of these points: (a) the excitation energy has to fit a chosen range of energies, and (b) the transition probability to the given excited state must be larger than the prescribed threshold value.

The excited-state dynamics is run with a smaller time step of 0.1 fs where the time-dependent Schrödinger equation
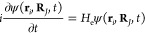
is solved in each step. Based on the evaluation of stationary states in the given step, the time-dependent wave function is used
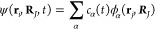
and finally, applying the bra-wave functions, a set of the equations for time-dependent coefficients *c*_α_(*t*) is found
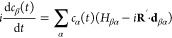
where the individual matrix elements can be evaluated:
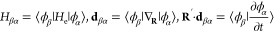


Symbol **d**_βα_ denotes the non-adiabatic coupling vector, **R**^′^ is the vector of the nuclear velocities, and **R**^′^ · **d**_βα_ is the time-derivative coupling term between the adiabatic electronic states α and β generated from the zero-order Hamiltonian.

The hopping probabilities between two states are determined according to Tully’s fewest switches algorithm^19,27^ so that the average normalized occupancies of the examined states over the whole swarm of trajectories is acquired. Finally, the time dependence of these occupancies (*S_x_*(*t*)) is fitted according to the decay law from a set of the ordinary differential equations
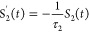
1
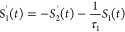
with initial conditions of *S*_2_(0) = 1 and *S*_1_(0) = 0 where the coefficients τ_2_ and τ_1_ represent averaged lifetimes of the two corresponding excited states.

For approximately half of the trajectories that reached the maximum simulation time, the S_1_ → S_0_ de-excitation step was located and the corresponding structures were used to start a conical intersection (CI) optimization at two different levels: (a) the OM2/MNDO level with the same settings of active space as in hopping dynamics and (b) the CASSCF(2,2)/6-31++G(d,p) computational model. From these calculations, several groups of CI structures with the same energy were obtained for each of the explored molecules, and their geometries were compared and analyzed. In order to draw molecular orbitals from the active space, another set of the ground-state CASSCF(8,8)/aug-cc-pVDZ level was performed, obtaining natural orbitals that were subsequently used for visualization. Program Gnuplot v 5.4.5^28^ is utilized for the fitting procedure and graph production, and VMD (visualized molecular dynamics^29^) and Gaussview 6.0 software are employed for visualization of the obtained structures and natural orbitals in the Supporting Information.

### QM/MM Dynamics with MNDO99 and MNDO2020 in Newton-X

Even though both MNDO99 and MNDO2020 contain an implementation of Tully’s surface hopping dynamics,^30^ the QM/MM approach that would allow adding a larger amount of explicit solvent molecules is not supported.

Newton-X is a specialized program for non-adiabatic dynamics that can use a large selection of third-party programs for quantum chemical calculations and also allows combining the use of several programs, employing the hybrid gradient approach.^31^

We have added a new interface to Newton-X that allows using MNDO99 and MNDO2020 in MD simulations. The interface can read energies, gradients, and non-adiabatic couplings in each step of the dynamics and prepare the necessary files in the Newton-X format. With this new interface in place, we are able to incorporate the MNDO calculations within the hybrid gradient approach. In this approach, the system is divided into any number of disjoint regions that can be treated at a different level of theory using different programs. Frequently, only two such disjoint regions are used. Typically, one region is treated by a method based on quantum mechanics (QM) while the other region is treated at a computationally cheaper molecular mechanics (MM) level. This combination, simply denoted as QM/MM, was also used in this work. In our simulations, the atoms in the QM region are calculated with MNDO2020 at the OM2/MNDO level. The rest of the system including the interactions between the QM and MM regions is calculated in Gromacs using (in our case) the SPCE force field.

The total energy of the system is based on Morokuma’s subtractive mechanism^32^
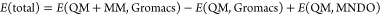
where the MM energy of the QM region *E*(QM, Gromacs) is replaced by the quantum-mechanically evaluated *E*(QM, MNDO) energy. An analogous relation holds for the energy gradients as well.

## Results and Discussion

First, a classification of the lowest excited states of the optimized structures is performed. From the single-point calculations, the dominant occupations and characters of MO are determined, which is important for assigning π–π* and *n*–π* characters to the individual transitions, and are summarized in Table 1. The frontier natural orbitals from CASSCF calculations of all the molecules are shown in Figure S1. For the discussion of the optimized geometries of the ground and first excited states, our previous study can be consulted. Some other details on geometry deformations in excited states are also discussed in the work of González-Luque et al.,^11^ Merchán et al.,^4^ Lan et al.,^33^ Shukla and Leszczynski,^34^ and Nachtigallova et al.^35^

**Table 1:** Comparison of Vertical Transition Energies Using the Time and Energy Averaged Spectra from the Trajectories at 298 K, Employing the OM2/MNDO Method with Experimental Data

		S_1_		S_2_		S_3_	
molecule	method	λ [nm]	*f*	λ [nm]	*f*	λ [nm]	*f*
*s*T	OM2/MNDO	284	0.006	265	0.002	257	0.000
	experiment	251		221			
	character	*n*–π*		*n*–π*		*n*–π*	
AT	OM2/MNDO	273	0.000	240	0.005	236	0.002
	experiment	262		221			
	character	*n*–π*		*n*–π*		π–π*	
DT	OM2/MNDO	283	0.055	234	0.004	227	0.000
	experiment	258		205			
	character	π–π*		*n*–π*		*n*–π*	
TT	OM2/MNDO	243	0.000	231	0.003	225	0.000
	experiment	241		206			
	character	π–π*		*n*–π*		*n*–π*	
5AC	OM2/MNDO	295	0.000	258	0.052	240	0.004
	experiment	250		226		205	
	character	*n*–π*		π–π*		*n*–π*	
Cyt	OM2/MNDO	286	0.065	264	0.000	236	0.064
	experiment	274	0.069	237	0.001	206	0.003
	character	π–π*		*n*–π*		*n*–π*	

Information on vertical excitation energies and absorption spectra of isolated molecules determined at various computational levels or using experimental methods can be found in refs (10) and (36) for Cyt, ref (37) for 5AC, and in refs (37c) and (38) for triazines.

### Lifetimes of the Excited States

**Figure 1:**
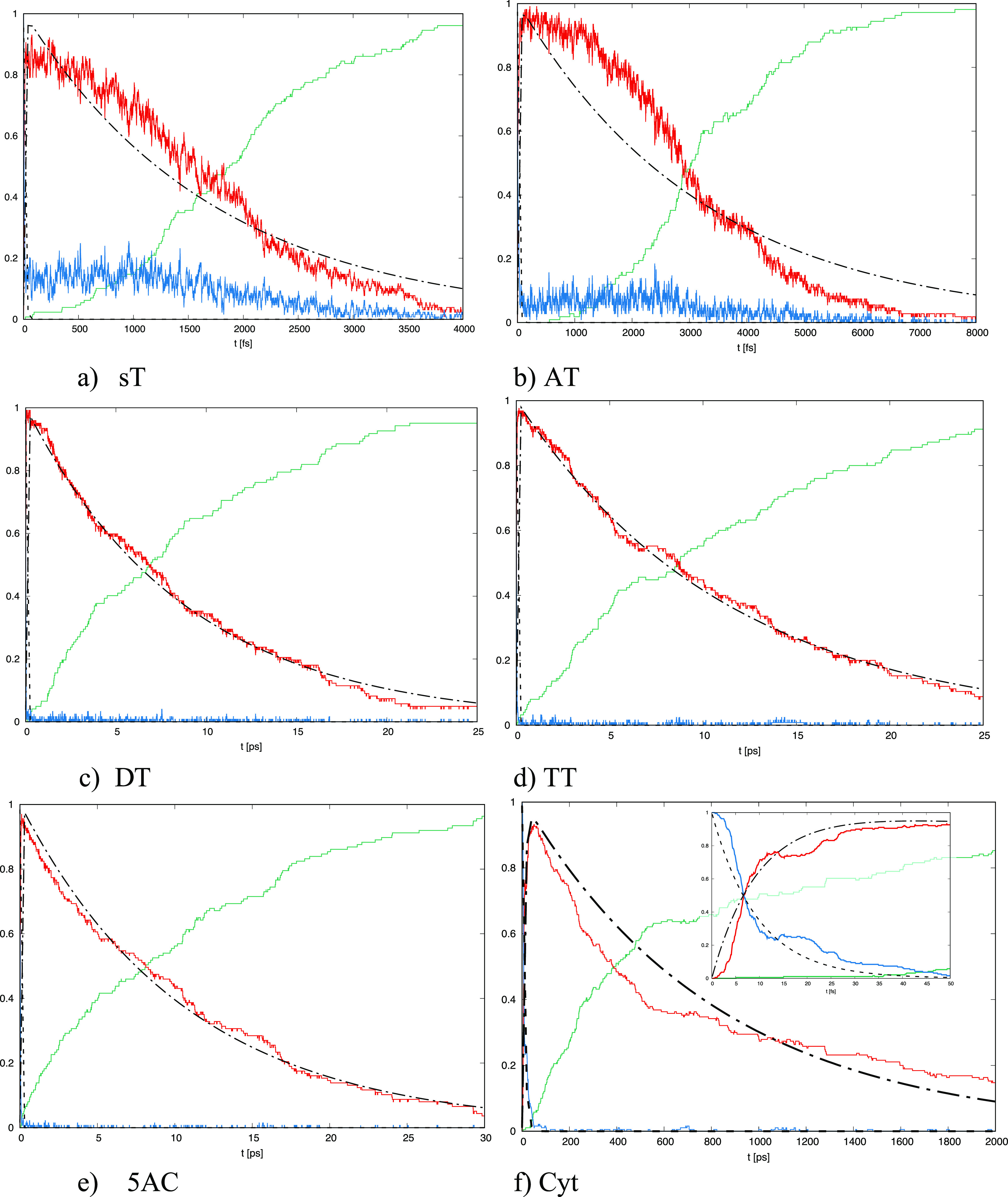
Dependence of the probability of the examined states on time for (a) *s*T, (b) AT, (c) DT, (d) TT, (e) 5AC, and (f) Cyt; the blue line corresponds to the second excited state (S_2_(t)), the red line to the first excited state (S_1_(t)), and the green line to the ground state (S_0_(t)). In the case of Cyt, the inset displays the first 50 fs of the time evolution.

As mentioned in the Computational Details, stochastic treatment is applied to approximately 200 hopping trajectories calculated for each molecule. The starting structures were selected from the ground-state MD trajectories. Contrary to previous gas-phase evaluations, relatively shorter ground-state MD trajectories are sufficient (up to 10 ps) within the QM/MM approach. In the hopping dynamics, most of the cytosine molecules end in the ground state before 2 ps. The S_1_ excited states of *s*T and AT last ca. 3–4 ps, and in the remaining three molecules (DT, TT, and 5AC), they stay excited substantially longer (up to 20 ps). The normalized abundance (probability) of individual occupied states as a function of time after excitation to the S_2_ state is displayed in Figure 1. By the fitting procedure of the solution of differential equations (eq 1), lifetimes τ_1_ and τ_2_ are collected in Table 2. From the table, it can be noticed that the S_2_ states de-excite after a very short time in all the explored molecules. All the lifetimes (τ_2_) are in the range of ca. 10–30 fs.

**Table 2:** Estimated Lifetimes τ_1_ and τ_2_ for the De-excitation of the S_1_ and S_2_ States, Respectively^e^

	no. of trj	τ_2_ [fs]	σ (fs)	τ_1_ [ps]	σ (ps)	τ_1_ [ps]exp
*s*T	343	7.3	0.5	1.9	0.04	
AT	246	11.3	0.7	4.1	0.12	
DT	174	10.4	0.7	10.6	0.55	17.0^c^
TT	183	11.7	0.9	13.1	0.79	13.0^c^^,^^d^
5AC	157	29.5	1.8	11.9	1.04	15.0^c^
Cyt	190	9.5	0.7	0.83	0.08	0.72^a^^,^^b^

a Ref (40).

b Ref (5).

c Ref (2).

d Ref (10).

e Explicit water in OM2/MNDO: cytosine with 20 water molecules, time step of 0.5 fs: . QMMM with QM: solute, MM: 100 water molecules using the classical (SPCE) force field, and Newton-X (MNDO2020 + Gromacs) with a time step 0.1 fs.

Regarding cytosine, Richter et al.^12b^ explored feasible deactivation pathways using a semi-classical ab initio method.^39^ They described the possibility of a transition from the S_2_ state directly to the ground state with a lifetime of ca. 25 fs. Fast deactivation of the S_2_ state is also confirmed by our calculations though we did not find any direct S_2_ → S_0_ hopping in the trajectories of our molecules neither in the gas phase in a previous study^20a^ nor in solution in the present dynamics. Nevertheless, Richter et al.^12b^ stated that this kind of de-excitation is rare. The dominant deactivation is realized in passing through the S_1_ state, estimating a lifetime of ca. 155 fs, which is a little bit too short. Lan et al.^33^ have found that τ_1_ = 370 fs, which is much closer compared to the experimental value and also to our simulations. Another way to verify the obtained lifetimes of solvated molecules is to directly include water molecules into the quantum description. Even though such simulations are much more time-demanding, we simulated another swarm of 180 surface-hopping dynamics where cytosine was placed into a box of additional 20 explicit water molecules. The received lifetime is noticeably longer (τ_1_ = 1.03 ps) than in the case of the QM/MM simulations. A possible explanation can be seen in the relatively small number of water molecules that cannot completely surround the cytosine molecule. Nevertheless, the Watson-Crick edge of cytosine is fully H-bonded usually by four water molecules in all the performed surface-hopping trajectories.

Considering the 5AC molecule, Borin et al.^17^ performed an ab initio MD simulation, arriving to a lifetime of τ_2_ = 32 fs for the transition from the S_2_ (π–π*) to S_1_ (*n*–π*) state and τ_1_ = 1.1 ps for the de-excitation to the ground state. However, the τ_1_ parameter seems to be too short in comparison with the corresponding values of the pyrimidine nucleobases evaluated in the same laboratory.^12b,15,16^ The 5AC molecule was explored using transient absorption spectroscopy by Zhou et al.^2^ They used a biexponential decay fit, obtaining lifetimes of τ_2_ = 1.5 ps and τ_1_ = 15 ps. The τ_1_ parameter is in reasonable accordance with our present result since our estimation is 11 ps.

In the same work of Zhou et al.,^2^ lifetimes of AT, DT, and TT molecules were determined too. For the relaxation mechanism of DT, a single-exponential decay was used with a lifetime of approximately 17 ps. This value is a little bit longer than the value determined in the present study: τ_1_ = 11 ps. Nevertheless, our simulations for the TT molecule agree with Zhou et al.’s^2^ results as well as with another experimental work, that is, the time-resolved IR measurement performed by Kohler and co-workers.^10^ The measured relaxation time is determined in both experiments to be approximately 13 ps, and our OM2/MNDO MD simulations in water suggest the same lifetime 13 ps (cf. Table 2). The most striking difference concerns the results obtained for the AT molecules. In contrast to the study of Zhou et al.^2^ and our gas phase results, the τ_1_ lifetime is estimated approximately 4ps, when evaluated either with 0.5 or 0.1 fs hopping dynamics based on more than 2× 200 trajectories. Lifetimes for the computational model with a 0.5 fs time step are included in Table S2. In the case of the triazine family, the QM/MM MD simulations in explicit water solution reveal that the fastest deactivation occurs for symmetrical 1,3,5-triazine with a τ_1_ lifetime of ca. 2 ps. Comparing *s*T, AT, DT, and TT molecules, their lifetimes increase with an increasing number of exocyclic amino groups. According to Zhou et al.,^2^ the higher the number of exocyclic amino groups that occurs in a molecule, the shorter the excitation time. Actually, we came to the same conclusion in our previous paper where gas-phase simulations were performed. Nevertheless, including explicit water molecules as a solvent of the examined molecules changed the obtained picture completely. According to our present simulations, the lifetime gets longer with the increasing number of exocyclic amino groups. From a more detailed analysis of individual trajectories, it is found that the easiest way to reach the conical intersection is by an out-of-plane movement of the carbon-bonded hydrogens. Indeed, the lowest-lying CI structures are really those where the hydrogen atom is oriented nearly perpendicular to the triazine plane (cf. discussion below). Such a situation is more probable when there are more carbon hydrogens present. It is more energetically demanding to “expel” the amino group to an out-of-plane position, especially when we take into account the “friction” forces of the surrounding water molecules. Using this picture, it is easy to explain the obtained trend of lifetimes increasing with the number of amino groups. This consideration can be also directly applied to the estimation of the lifetime of 5-azacytosine since, in this molecule, there is only one carbon-bonded hydrogen available for the out-of-plane movement in order to form the energetically low-lying CI structure.

**Figure 2:**
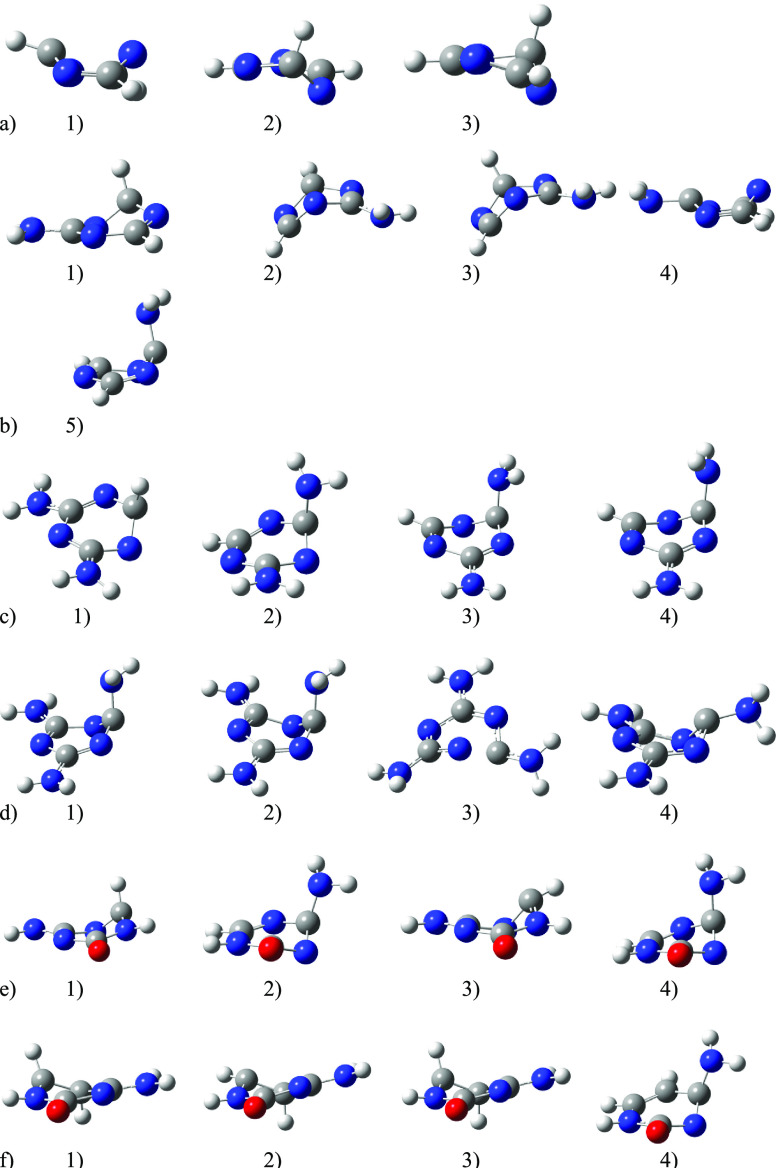
Structures of conical intersections obtained with CASSCF(2,2)/6-31++G(d,p) (ordered by increasing energies as summarized in Table 3): (a) *s*T, (b) AT, (c) DT, (d) TT, (e) 5AC, and (f) Cyt.

**Figure 3:**
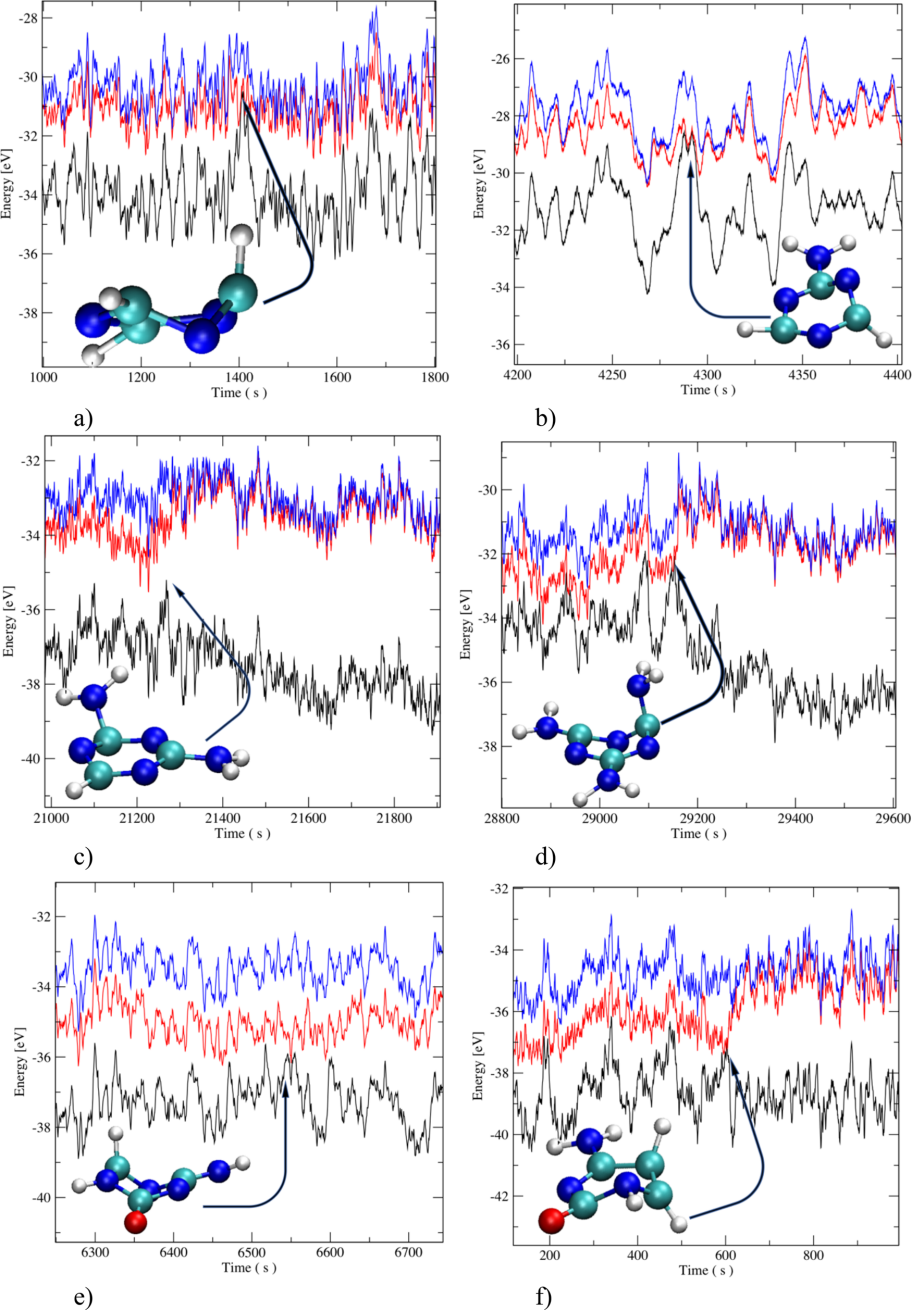
Fragments of the trajectories of examined molecules with an inset of the structures when de-excitation to the ground state occurs: (a) *s*T, (b) AT, (c) DT, (d) TT, (e) 5AC, and (f) Cyt. The black line is the ground-state energy, the red line is the first excited state, and the blue line is the second excited state.

A typical MD trajectory for each of the examined molecules is depicted in Figure 3 together with an inset of the CI structures. After this point, the ground state is populated and the molecule starts to slowly decrease its energy because of the interaction with thermostated solvent molecules. From the individual trajectories, it follows that the de-excitation predominantly occurs when both the first excited and ground states are practically degenerated. Nevertheless, it can be also often noticed that a small energy gap exists between both states, which is usually up to 1 eV. Despite this gap, the tested hopping probability is sufficiently high to allow the transition in the surface hopping algorithm.

Since only very tiny standard deviations were obtained from the τ_1_ and τ_2_ fitting procedure of the normalized averaged populations, another way for estimation of the statistical error was suggested. In analogy with a stochastic walk, the complete set of trajectories was divided into smaller parts (two-halves, three-thirds, etc.) till more than 30 trajectories remained in such a smaller set. The order of the complete set of trajectories was reshuffled before each division so that not all trajectories contained, for example, in the first third were also in the first half. Then, a pair of lifetimes τ_1_ and τ_2_ was determined for each of such divisions. This procedure was repeated 10 times and for each subset (halves, thirds, quarters, ...) averaged. Finally, the standard deviations were determined from the fact that these partial deviations exhibit 1/*N* dependence (where *N* was the number of trajectories in the given subset). The least-squares fitting of these partial deviations on the 1/*N* behavior enabled us to extrapolate to the limit of the complete number of trajectories and thus determined the standard deviation for it.

### Conical Intersections

From the potential energy profiles of the explored trajectories, structures of the examined molecules were extracted in the MD step when the deactivation from the first excited state to the ground state occurred. More than a hundred structures for each of the examined molecule were chosen for the conical intersection optimizations using the same OM2/MNDO computational model and also at the CASSSCF(2,2)/6-31++G(d,p) level.

The resulting energies are summarized in Table 3 for both CASSCF and OM2/MNDO methods together with energies of the first excited state in the optimal ground-state geometries. Here, it can be noticed that the energy of the first excited state in the S_0_ geometry increases with the number of amino groups starting from *s*T. The 5AC molecules have a lower excitation energy than all the triazines. The only inconsistency between both computational methods concerns cytosine. While, in CASSCF calculations, it has the lowest excitation energy, in the OM2/MNDO approach, its S_1_ energy is higher than the value obtained for the 5AC molecule. Also, except for cytosine, all S_1_ energies at the OM2/MNDO level are lower than the CASSCF energies.

**Table 3:** Energies of Conical Intersections at the CASSCF and MNDO Levels^a^^,^^b^

	CASSCF(2,2)/6-31++(d,p)						OM2/MNDO			
	CI1	CI2	CI3	CI4	CI5	S1/S0	CI1	CI2	CI3	S1/S0
*s*T	4.28	4.31	4.72			5.26	4.98	5.24		4.56
AT	4.28	4.37	4.58	4.67	4.72	5.40	4.64	4.73	4.88	4.67
DT	4.33	4.67	4.76	4.92		5.48	4.63	4.71	4.83	4.97
TT	4.26	4.58	4.82	4.85		6.21	4.89	5.37		5.14
5AC	3.82	4.07	4.20	4.26		5.17	4.15	4.24	4.28	4.33
Cyt	3.33	3.45	3.53	3.82		4.20	3.49	3.74		4.58

a Cyt: cas_3 has the largest torsion angle: 147 vs 126 for cas_1.

b *s*T: cas_2 and cas_3 differ in the torsion angle: 95 vs 80.

Regarding the energies of the conical intersections, they are all lower than the energies of vertical excitations in the optimal ground state (GS) structures. However, both of the lowest CI energy OM2/MNDO values of the *s*T molecule are already above the S_1_ excitation energy at the GS geometry. In the AT and TT molecules, the first CI energies are lower than the excitation energy in the GS geometry. However, the remaining values are already higher.

In Figure 2, several lowest CI structures optimized at the CASSCF level are depicted. From this figure, it can be seen that, in most of the structures, one of the “exocyclic ligands” (either hydrogen or the NH_2_ group) is visibly pointing nearly perpendicular to the heterocyclic ring, which is also substantially deformed. There are only very few exceptions: (a) the lowest CI energy of the *s*T molecules and fifth CI structure of AT have a boat-like shape; (b) the third CI structure of AT has a book-like form of the six-membered ring, and a nearly book-like form exists also in the fourth TT structure where the amino groups bonded to the neighboring carbon atoms probably do not allow bending of the C–N–C bonds enough while keeping the sp^2^ hybridization; and (c) the third TT structure has a relatively long N–C bond (1.6 Å). Therefore, the closest amino group lies approximately in the molecular plain and not perpendicularly. Although the pictures of Cyt, namely, c1 and c3, look similar, there is a difference in the torsion angle (N1–C6–C5–H5) of 126° versus 147° systematically for more than 20 optimized cases from each c1 and c3 group (not averaged) (cf. Computational Details). Simultaneously, the whole ring in c3 is more deformed. A similar situation occurs in *s*T with the structures for the a2 and a3 conformers and the first two lowest TT structures d1 and d2. These three pairs of similar structures form quite an energetically distinguished group so that they are really different conformations. The *xyz* coordinates of all the displayed CI-optimized structures are available in the Supporting Information.

Concerning the MNDO CI optimization, usually, the received structures are very similar and their geometries are displayed in Figure S2 of Supporting Information where the individual *xyz* coordinates are also included. However, some basic distinctions exist too. The most striking fact is that, in several cases, the order of energies is different for both methods used. In the *s*T molecule, the CASSCF a1 structure was not found so a2 is the lowest-lying conformer at the MNDO approach followed by a nearly planar form with a broken N–C bond similar to the CASSCF d3 form of the TT molecule. Nevertheless, the N···C distance is visibly longer (2.2 Å). In the case of AT, besides the different order of CI structures, the lowest structure has also a broken ring between the C1 and N2 atoms with a distance of 2.1 Å (C1 labels the carbon where the amino group is attached). Further, the b5 CAS structure is lower than the b1 form. As for DT, the first two structures have an inverted energy order. Interestingly, the MNDO c3 boat-like form is not found among the explored CASSCF structures. Concerning the TT conical intersection structures, the MNDO approach did not find the d1 and d2 forms mentioned in the previous paragraph. Probably, the ring deformations connected with these structures caused the C–N bond breakage since, under the presence of the three electron-withdrawing NH_2_ groups, the ring C–N bond can be slightly destabilized. Nevertheless, in searching for this effect from the bond critical points in QTAIM analysis, only minor changes were found to confirm this fact. In the 5AC molecule, the energy preferences of the first two forms are also exchanged compared to CASSCF and the third MNDO CI structure (e3) has a visibly more deformed six-membered ring compared to the slightly lower-lying e2 form. Finally, in cytosine, only two distinct structures are within the 5 eV range above the ground state. The lowest one is close to the CASSCF f1 cytosine shape, and the other form is close to the f4 CASSCF structure. Note that our CI structures of cytosine can be compared with analogous structures presented for this molecule in Lan et al.’s study^33^ where also the remaining pyrimidine nucleobases were explored.

Another interesting feature of the triazine CI structures is the boat-like form, which occurs for *s*T and AT only in CASSCF optimizations, and even the MNDO reoptimization starting from CASSCF structures does not reproduce this shape. On the contrary for the DT molecule, this shape was not seen among the CASSCF results from the chosen set of hopping trajectories but exists in OM2/MNDO results. Similarly, starting from the OM2/MNDO CI structure, the CASSCF reoptimization ended again in the c1 form where no boat-like shape is present. This demonstrates that the energy surfaces of the excited states can deviate from ab initio ones. Nevertheless, many studies point to the fact how delicate of a task is the correct prediction of the order of the excited states or structures of conical intersections.

## Conclusions

To explore the de-excitation dynamics, a swarm of semi-classical MD simulations is performed where the time-dependent Schrödinger equation is solved in each step of the trajectories together with the Tully’s fewest switches algorithm. By applying this stochastic treatment, probabilities of individual states in time are determined and, from a biexponential fit, the lifetime parameters are determined. The obtained values are compared with other studies of these molecules with either experimental or computational techniques. The generally known fact that nucleobases are relatively quickly deactivated is confirmed in the cytosine case where approximately 80% of molecules ended in the GS below 2 ps (our best estimation is τ_1_=0.82 ps using a QM/MM hybrid approach with 100 water molecules treated with the empirical SPCE force field and a time step of 0.1 fs). Longer lifetimes are obtained for the remaining molecules where 5AC, DT, and TT de-excite predominantly within the first 20 ps. The increasing τ_1_ lifetime in the triazine family (*s*T < AT < DT < TT) with the increasing number of exocyclic amino groups can be explained by a higher mobility of the carbon-bonded hydrogen atoms in comparison with heavier amino groups where their movement is comparatively more slowed down by the surrounding water environment.

Comparing cytosine (or generally pyrimidine nucleobases) with the remaining set of molecules, it follows that, besides the number of carbon-bonded hydrogen atoms (more or less, the mechanical criterium), the other important factor relates to the energies of the CI structures. These energies are visibly lower in comparison with triazines or 5AC (below 3.5 eV predicted by both the CASSCF and MNDO methods), and maybe it is partially connected also with a lower HOMO–LUMO gap (cf. Table S1).

Finally, it can be concluded that both targets of this paper have been successfully fulfilled and, in comparison with our previous study, the role of explicit treatment of solvent molecules has been clearly elucidated. The main role of the solvent molecules is to enforce structural and consequently also electronic deformations mainly due to their mechanical influence. This causes larger deformations and especially the activation of the movement of the carbon-bonded hydrogen atoms, which makes some of the CI structures easier to access. This holds especially for the relatively rigid molecules such as 1,3,5-triazine, which did not de-excite without the presence of an explicit solvent in any of over 200 OM2/MNDO surface-hopping MD trajectories.^20a^

## Supporting Information Available

Frontier MOs of pyrimidine nucleobases and the triazine family (in Hartree), estimated lifetimes τ_1_ and τ_2_ for de-excitation of the S_1_ and S_2_ states in MD simulations with 0.5fs time-step, natural orbitals of *s*T, AT, DT, TT, 5AC, and Cyt molecules obtained at the CASSCF(8,8)/aug-cc-pVDZ level, and structures of conical intersections obtained with the OM2/MNDO method (PDF)
