# Supplementary material for: Vertical Excitation Energies and Lifetimes of the Two Lowest Singlet Excited States of Cytosine, 5-Aza-cytosine, and the Triazine Family: Quantum Mechanics–Molecular Mechanics Studies
Source: J Chem Theory Comput. 2023 Mar 24;19(7):1976–85. doi: 10.1021/acs.jctc.2c01262 (PMC10100535; doi:10.1021/acs.jctc.2c01262)
Supplement: Supplementary file 1 — ct2c01262_si_001.pdf [file ct2c01262_si_001.pdf]

Vertical excitation energies and lifetime of the two lowest singlet excited states of cytosine, 5-aza-cytosine, and triazine family; QM/MM studies

Ondřej Tichý,[a] Marek Pederzoli,[b] Jiří Pittner,[b] and Jaroslav V. Burda[a]

[a]*Department of Chemical Physics and Optics. Faculty of Mathematics and Physics. Charles University. Ke Karlovu 3. 121 16 Prague 2. Czech Republic*

[b]*J. Heyrovský Institute of Physical Chemistry, Academy of Sciences, Dolejškova 3, 182 23 Prague 8, Czech Republic*

Supplementary material

Table S1 Frontier MOs of pyrimidine nucleobases and triazine family (in Hartree); HO-LU means HOMO/LUMO energy gap. Bold is used for labelling π character of MOs.

MO:	HOMO-2	HOMO-1	HOMO	LUMO	LUMO+1	HO-LU	π–>π*
sT	**-0.445**	-0.445	-0.445	**0.070**	**0.076**	-0.515	-0.515
AT	-0.437	-0.430	**-0.374**	**0.063**	**0.083**	-0.437	-0.437
DT	-0.420	**-0.386**	**-0.361**	**0.064**	**0.074**	-0.425	-0.425
TT	**-0.401**	**-0.363**	**-0.363**	0.065	**0.077**	-0.428	-0.440
5AC	-0.425	-0.410	**-0.381**	**0.050**	0.065	-0.432	-0.432
Cyt	**-0.421**	-0.386	**-0.345**	**0.049**	0.061	-0.394	-0.394
Thy	-0.450	**-0.436**	**-0.354**	**0.046**	0.069	-0.400	-0.400
Ura	**-0.452**	-0.439	**-0.370**	**0.043**	0.074	-0.413	-0.413

Table S2: Estimated lifetimes τ_1 and τ_2 for deexcitation of the S_1 and S_2 states in MD simulations with 0.5fs time-step.

	# trj	τ_2 [fs]	σ (fs)	τ_1 [ps]]	σ (ps)	τ_1 [ps]exp
sT	129	10.6	1.3	1.7	0.07	-
AT	188	16.4	1.4	3.0	0.10	-
DT	122	12.8	1.1	8.9	0.63	17.0 [c)]
TT	125	15.0	1.5	11.4	0.90	13.0 [c,d)]
5AC	137	31.6	2.2	10.8	0.71	15.0[c)]
Cyt	186	12.3	0.8	0.55	0.04	0.72[a)]

[a)] ref. [41], [b)] ref. [5], [c)] ref. [2], [d)] ref. [11]

QMMM – QM: solute + MM: 100 water molecules using classical (SPCE) force field: Newton-X (MNDO2020 + Gromacs) with timestep 0.5fs.

Cation of Figures:

Figure S1 Natural orbitals of sT, AT, DT, TT, 5AC, and Cyt molecules obtained at the CASSCF(8,8)/aug-cc-pVDZ level.

Figure S2. Structures of conical intersections obtained with OM2/MNDO method (ordered by increasing energies):
a) sT, b) AT, c) DT, d) TT, e) 5AC, and f) Cyt

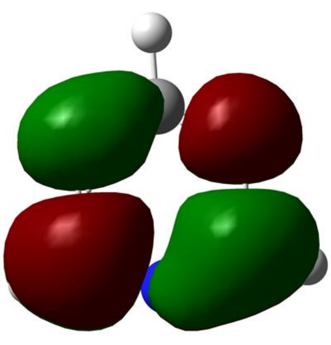

LUMO+1

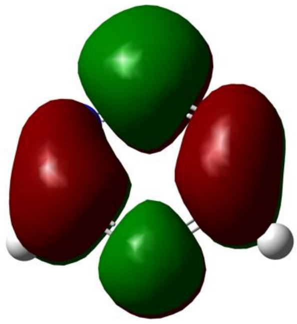

LUMO

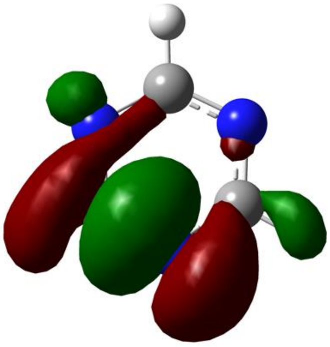

HOMO

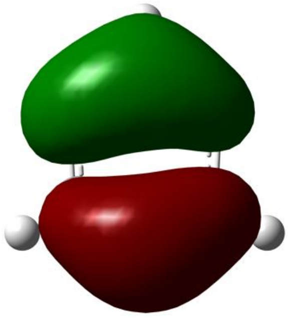

HOMO-1

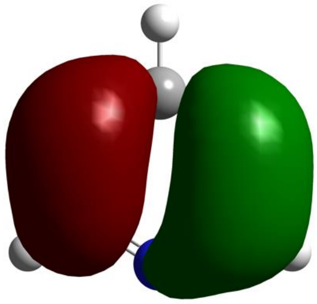

HOMO-2

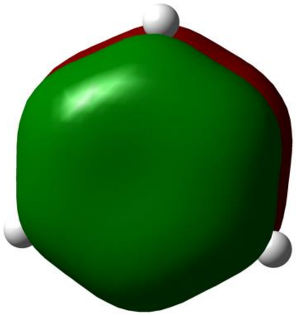

HOMO-3

1,3,5-triazine

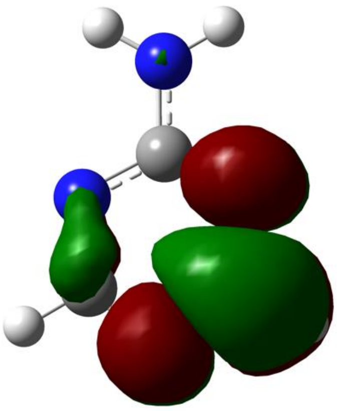

LUMO+1

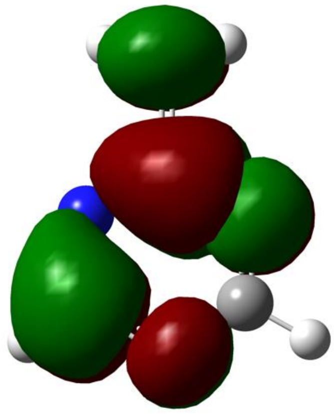

LUMO

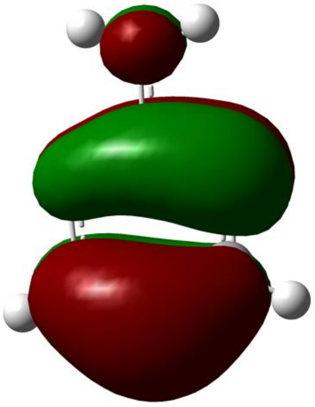

HOMO

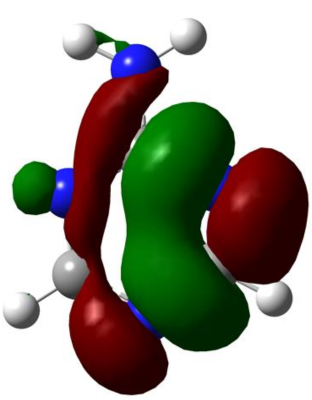

HOMO-1

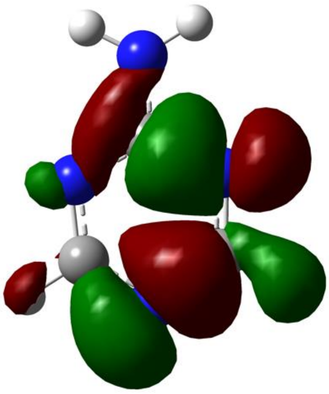

HOMO-2

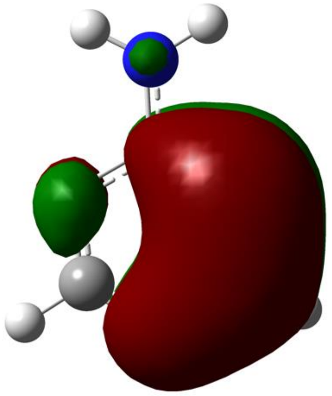

HOMO-3

2-amino-1,3,5-triazine

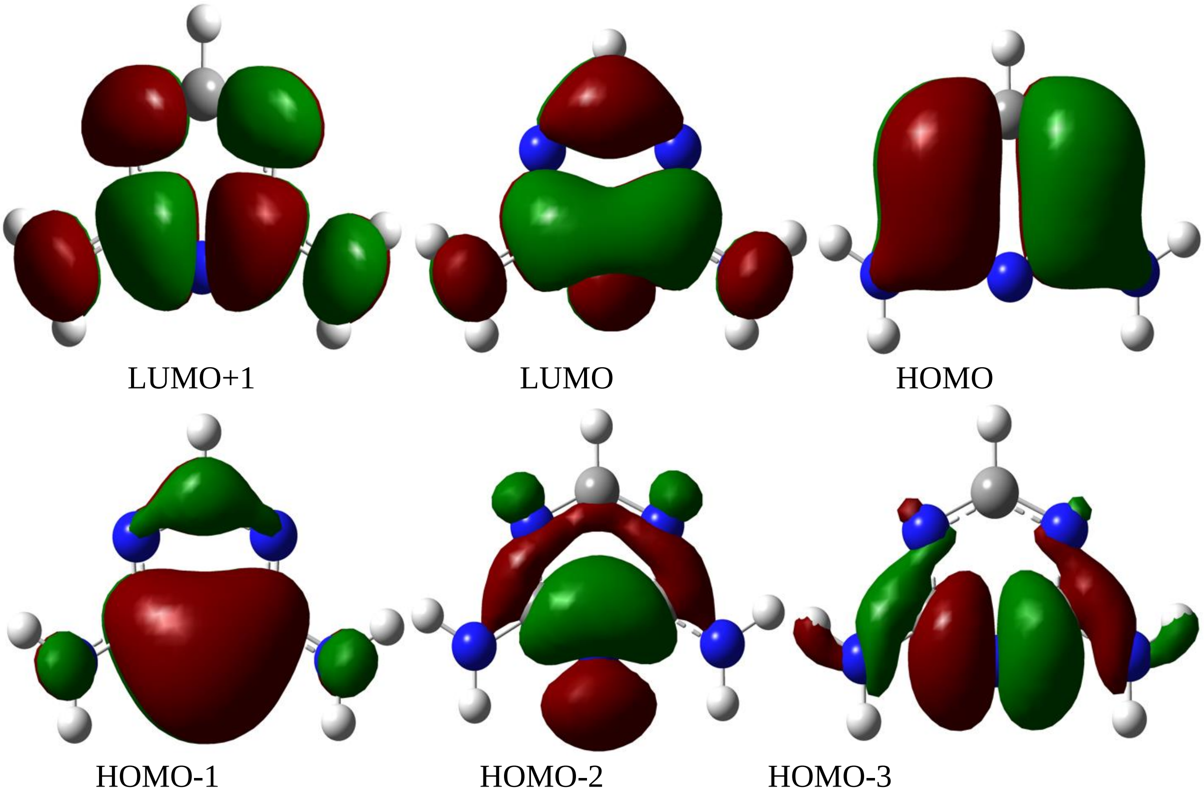

2,4-diamino-1,3,5-triazine

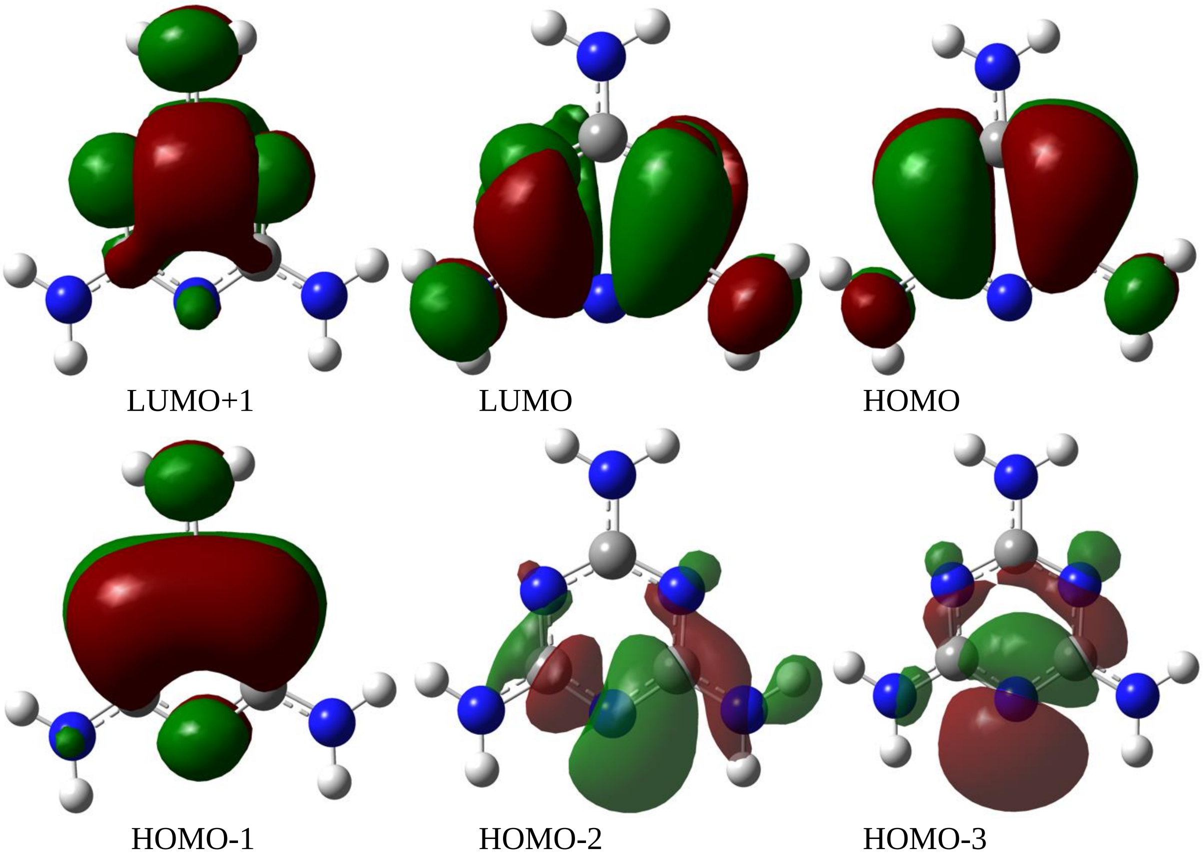

2,4,6-triamino-1,3,5-triazine

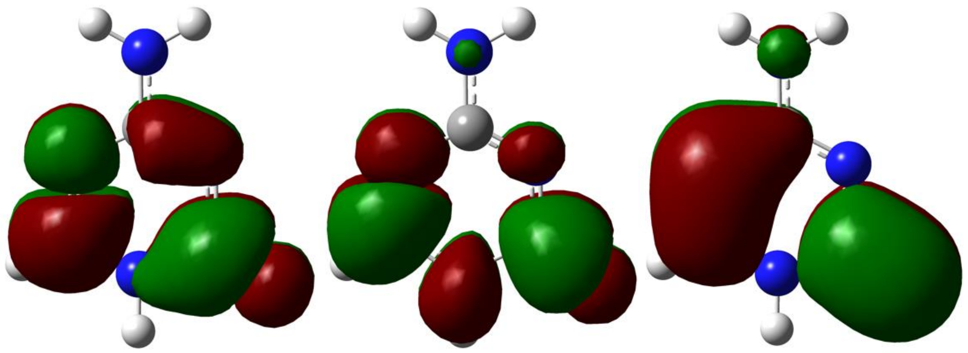

LUMO+1 LUMO HOMO

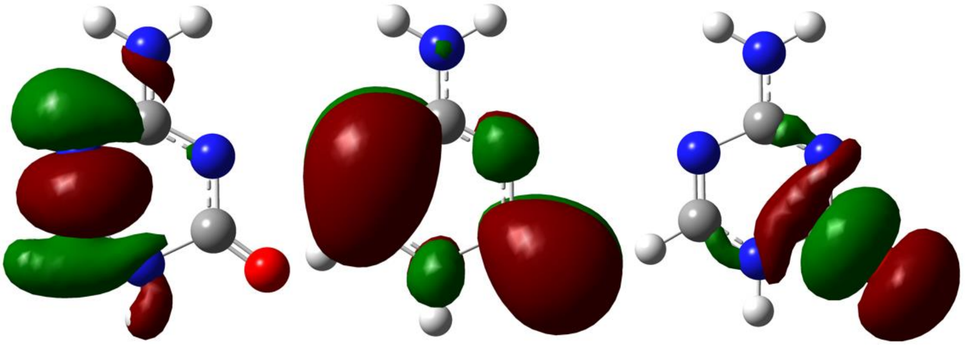

HOMO-1 HOMO-2 HOMO-3

5-azacytosine

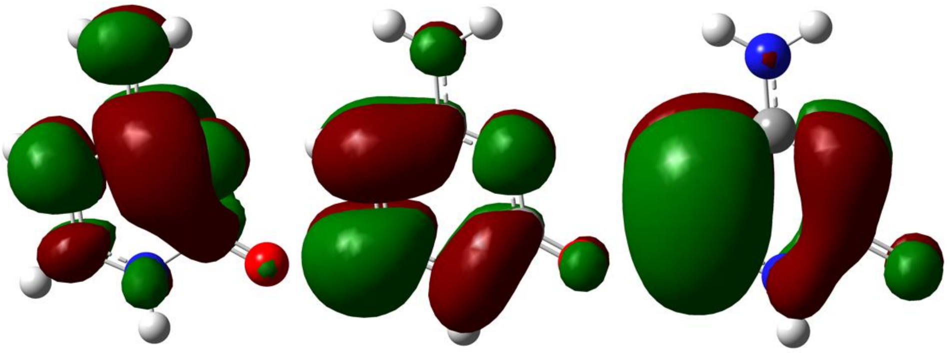

LUMO+1 LUMO HOMO

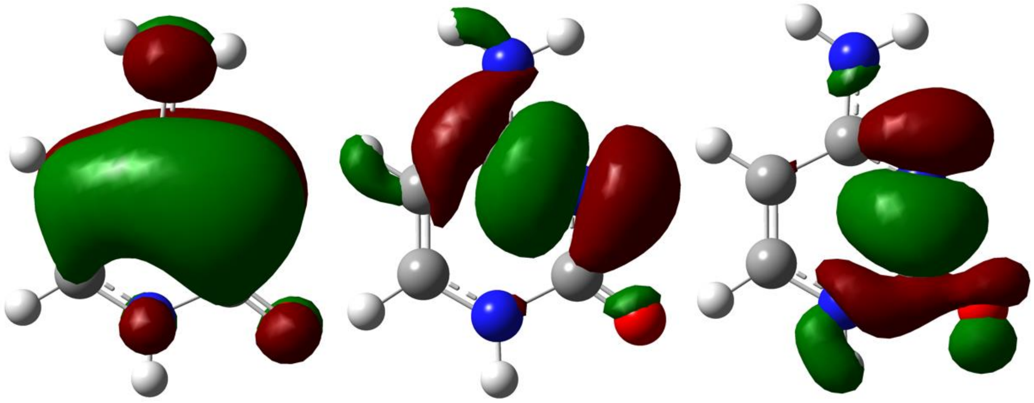

HOMO-1 HOMO-2 HOMO-3

Cytosine

Figure S1

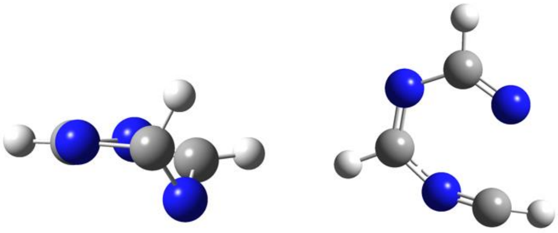

a) 1) 2)

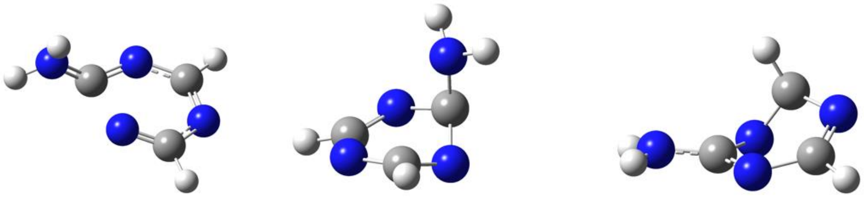

b) 1) 2) 3)

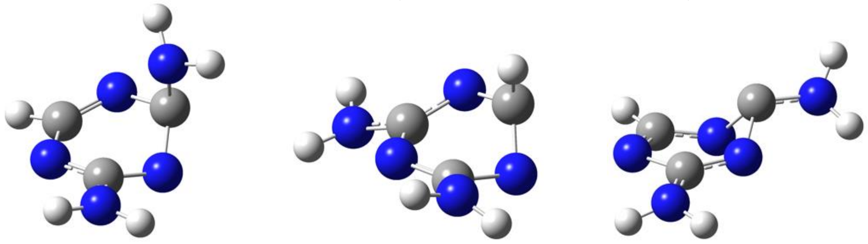

c) 1) 2) 3)

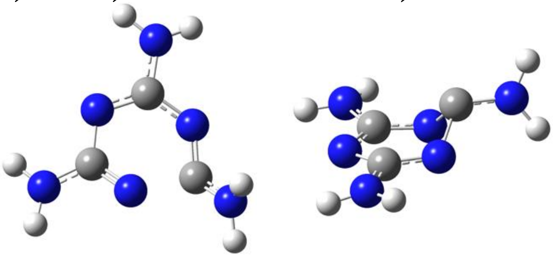

d) 1) 2)

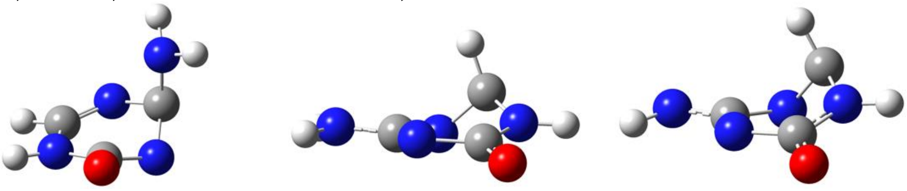

e) 1) 2) 3)

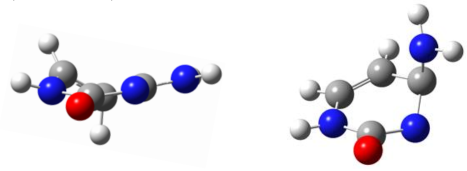

f) 1) 2)

Figure S2

XYZ coordinates of CI structures

Part 1 Optimized with the CASSCF/6-31++G(d,p) level of theory

sT:

```
1) 4.28 eV r_sT.run0095
   C   -1.155982   -0.618100   -0.152259
   H   -1.897270   -1.297448   -0.531594
   N    0.000086   -1.149234    0.498624
   C    1.155992   -0.617979   -0.152258
   H    1.897336   -1.297220   -0.531647
   N    1.218997    0.626254   -0.214139
   C   -0.000129    1.177386    0.311082
   H   -0.000151    2.224462    0.531604
   N   -1.218969    0.626175   -0.214163

2) 4.31 eV r_sT.run0033
   C   -0.462881   -1.246515    0.222493
   H   -0.404614   -1.509109    1.273688
   N   -1.377762   -0.197762   -0.069000
   C   -0.833118    0.937669   -0.108088
   H   -1.428039    1.809121   -0.310731
   N    0.534606    1.206042    0.169837
   C    1.291176    0.200927    0.072800
   H    2.345286    0.264244    0.283493
   N    0.774056   -0.996386   -0.439363

3) 4.72 eV r_sT.run0069
   C   -0.833008   -0.910080   -0.104239
   H   -1.405345   -1.794206   -0.318024
   N   -1.398882    0.222050   -0.110174
   C   -0.422463    1.181920    0.309805
   H   -0.291814    1.211073    1.389233
   N    0.706212    0.975288   -0.470178
   C    1.315571   -0.170094    0.042373
   H    2.380756   -0.247655    0.172292
   N    0.543498   -1.165866    0.190191
```

AT:

```
1) 4.28 eV r_AT.run0089
   C    1.199428   -0.994823   -0.079146
   H    1.748345   -1.906289   -0.235216
   N   -0.180138   -1.168328    0.059729
   C   -0.864415   -0.091865   -0.053950
   N   -2.182039   -0.021691    0.150981
   H   -2.687962    0.766315   -0.179917
   H   -2.672420   -0.867472    0.334083
   N   -0.220054    1.076329   -0.497151
   C    0.990116    1.201095    0.255426
   H    0.859476    1.447565    1.304929
   N    1.839629    0.095610    0.006747

2) 4.37 eV s_AT.run0059
   C    1.105149    1.032388    0.259860
   H    1.695281    1.918233    0.372245
   N   -0.270684    1.219839   -0.076426
```

C	-0.874058	0.128538	0.078288
N	-2.151465	-0.163708	-0.261276
H	-2.621339	-0.849700	0.285435
H	-2.703900	0.613099	-0.550119
N	-0.111410	-0.946115	0.628147
C	1.059690	-1.108963	-0.187431
H	1.264580	-2.085315	-0.586824
N	1.765087	-0.096887	-0.351165

3) 4.58 eV AT_CI0

C	-1.081072	1.110591	0.199803
C	0.873397	0.108398	0.051542
C	-1.131697	-1.061388	-0.170155
N	0.312411	1.214374	-0.129109
N	0.093129	-0.970955	0.563912
H	-1.428450	-2.042204	-0.497711
H	-1.356712	1.348702	1.214782
N	-1.785034	-0.021823	-0.363358
N	2.163730	-0.206366	-0.225385
H	2.579997	-0.923868	0.324120
H	2.751749	0.565141	-0.450756

4) 4.67 eV r_AT.run0017

C	-1.070433	1.154348	-0.114545
H	-1.778362	1.901395	-0.422797
N	0.161648	1.216844	-0.295042
C	0.779090	0.000108	0.165129
N	2.158735	0.000175	0.068986
H	2.588228	0.830382	0.414698
H	2.587899	-0.831290	0.412055
N	0.161730	-1.216874	-0.294884
C	-1.070299	-1.154455	-0.114470
H	-1.778203	-1.901619	-0.422563
N	-1.546357	0.000017	0.578357

5) 4.72 eV AT_CI1

C	-0.939361	1.086273	-0.103500
C	0.932741	0.072800	0.481739
C	-0.859499	-1.172407	-0.109968
N	0.181458	1.295452	0.456611
N	0.067105	-0.923130	0.887774
H	-1.170634	-2.169856	-0.364525
H	-1.592867	1.923227	-0.273986
N	-1.448663	-0.146849	-0.575243
N	1.697607	-0.163130	-0.675418
H	2.017853	0.679366	-1.105887
H	2.459804	-0.789136	-0.521296

DT:

1) 4.33 eV r_DT.run0091

C	-0.038502	1.697881	-0.224430
H	-0.335977	1.938987	-1.241362
N	-1.033781	1.000906	0.546002
C	-1.106364	-0.307062	0.051129
N	-2.325385	-0.825319	-0.129141

```
   H   -2.395210   -1.792439   -0.349467
   H   -3.111960   -0.365304    0.266072
   N   -0.024655   -0.961692   -0.147568
   C    1.140784   -0.195909   -0.017881
   N    2.249784   -0.945798    0.138987
   H    3.129673   -0.491833    0.064221
   H    2.188981   -1.919889   -0.037303
   N    1.212464    1.083477   -0.059005
```

2) 4.67 eV r_DT.run0024

```
   C    0.528020    1.568254   -0.106548
   H    0.730188    2.608796   -0.291423
   N   -0.786017    1.192403   -0.347475
   C   -1.086612    0.017709    0.072784
   N   -2.243773   -0.580701   -0.196414
   H   -2.498368   -1.414655    0.277331
   H   -2.915006   -0.098974   -0.748905
   N   -0.214729   -0.654262    0.944903
   C    1.139153   -0.530271    0.443785
   N    1.392537   -1.367682   -0.658490
   H    1.568477   -2.316903   -0.401767
   H    2.132496   -1.015143   -1.228969
   N    1.494674    0.824917    0.247992
```

3) 4.76 eV r_DT.run0032

```
   C   -0.310439    1.552485   -0.144009
   H   -0.630890    2.515916   -0.502344
   N    0.820763    1.091177   -0.459240
   C    1.062658   -0.219667    0.018324
   N    2.363809   -0.550091   -0.019502
   H    2.621856   -1.487140    0.183272
   H    2.996050    0.043569   -0.500705
   N    0.171204   -1.042889    0.413300
   C   -1.139852   -0.530663    0.457234
   N   -1.951649   -0.852841   -0.651931
   H   -1.669542   -1.716630   -1.066620
   H   -2.925525   -0.877716   -0.428936
   N   -1.127862    0.884512    0.763951
```

4) 4.92 eV r_DT.run0030

```
   C    0.317619    1.550208    0.127819
   H    0.643983    2.515326    0.478049
   N   -0.811096    1.094304    0.459323
   C   -1.064392   -0.219755   -0.014295
   N   -2.370444   -0.535010    0.020241
   H   -2.635766   -1.471484   -0.176727
   H   -2.997541    0.065179    0.500051
   N   -0.179665   -1.053609   -0.395483
   C    1.133273   -0.554156   -0.454945
   N    1.979740   -0.957333    0.579989
   H    2.903596   -0.588854    0.576898
   H    1.581339   -1.084926    1.485601
   N    1.122235    0.866931   -0.780547
```

TT:

1) 4.26 eV r_TT2.run0018

```
   C   -0.270461    1.370954   -0.425312
   N   -0.824462    1.999357    0.712309
   H   -0.115074    2.439968    1.260084
   H   -1.547414    2.651857    0.488386
   N   -1.103139    0.278903   -0.900541
   C   -0.896014   -0.843090   -0.087634
   N   -1.975455   -1.559148    0.221454
   H   -2.853962   -1.335618   -0.181686
   H   -1.866256   -2.395941    0.745704
   N    0.289022   -1.188550    0.257574
   C    1.266900   -0.238871   -0.020201
   N    2.519081   -0.724977    0.080347
   H    2.649674   -1.617917    0.491557
   H    3.274481   -0.081769    0.050928
   N    1.074381    0.995195   -0.322013

2) 4.58 eV TT.run0186
   C   -0.191168    1.355668   -0.416224
   C   -0.968498   -0.739662   -0.092972
   C    1.240027   -0.282479   -0.017471
   N   -0.966395    0.326553   -0.985861
   N    0.188261   -1.131705    0.324390
   N    1.153114    0.953897   -0.404726
   N   -0.703647    1.887824    0.787562
   H   -1.431230    2.556893    0.645716
   H    0.015825    2.269678    1.365632
   N   -2.093206   -1.394140    0.171982
   H   -2.954185   -1.099467   -0.222070
   H   -2.053617   -2.218434    0.725350
   N    2.445919   -0.848049    0.132509
   H    2.514717   -1.769796    0.489592
   H    3.258006   -0.300695   -0.025207

3) 4.82 eV r_TT2.run0003
   C   -1.254823    0.500693   -0.333319
   N   -2.502499    1.046895   -0.184084
   H   -2.567736    2.021028   -0.375281
   H   -2.974549    0.777174    0.655621
   N   -0.065759    1.177437    0.575344
   C    1.103662    0.728623    0.069557
   N    2.088963    1.611831   -0.119435
   H    2.035725    2.517591    0.282863
   H    2.974034    1.260359   -0.402911
   N    1.271118   -0.532212   -0.227937
   C    0.125668   -1.252916   -0.026244
   N    0.302577   -2.567289    0.070283
   H    1.219589   -2.943264    0.034855
   H   -0.490127   -3.156320    0.168393
   N   -1.100682   -0.784516    0.082471

4) 4.85 eV r_TT2.run0050
   C   -1.280442    0.223889   -0.265658
   N   -2.602008    0.676623   -0.207090
   H   -3.017815    0.483107    0.686318
   H   -3.147697    0.239001   -0.920321
   N   -0.317166    1.025671    0.651484
   C    0.907188    0.945686    0.057713
   N    1.639999    2.041228   -0.103758
```

```
H    1.314741    2.926245    0.205394
H    2.549397    1.954154   -0.494051
N    1.337267   -0.234867   -0.290917
C    0.391436   -1.201294   -0.024489
N    0.869502   -2.435603    0.057028
H    1.845641   -2.592211   -0.025033
H    0.240876   -3.188145    0.212079
N   -0.912483   -1.020457    0.140428
```

5AC:

```
1) 3.82 eV 5AC_CI8
   C    0.115247    1.673171    0.239061
   C    1.187944   -0.468607   -0.012198
   C   -1.100865   -0.264964   -0.045911
   N    1.258431    0.920126   -0.059782
   N   -0.086446   -1.024333    0.097406
   N   -2.356428   -0.692818    0.125687
   O    2.177771   -1.129005   -0.053631
   H   -3.108672   -0.152887   -0.233854
   H   -2.494722   -1.665386    0.283340
   H    2.165213    1.305460    0.081416
   H   -0.082306    1.950935    1.269025
   N   -0.974932    1.076498   -0.457110

2) 4.07 eV 5AC_CI1
   C   -0.250259    1.619715   -0.090444
   C    1.171293   -0.311460    0.077614
   C   -1.170520   -0.382917    0.434402
   N    0.990658    1.024371   -0.226630
   N    0.136938   -0.758526    0.918941
   N   -1.626521   -1.167496   -0.635744
   O    2.111535   -0.950000   -0.255735
   H   -1.795782   -2.119309   -0.382499
   H   -2.433703   -0.768873   -1.068222
   H    1.734790    1.496444   -0.690004
   H   -0.272666    2.676489   -0.292267
   N   -1.305077    1.010683    0.221924

3) 4.20 eV r_5AC.run0061
   H    2.176895    1.263044   -0.134112
   N    1.254562    0.896025   -0.044753
   C    0.170383    1.590388    0.427431
   H    0.203835    2.653510    0.558275
   N   -0.838627    1.033339   -0.529646
   C   -1.118807   -0.237955   -0.044374
   N   -2.395555   -0.601921    0.094588
   H   -2.579970   -1.559653    0.290865
   H   -3.118966   -0.046821   -0.298540
   N   -0.135294   -1.026639    0.201315
   C    1.143963   -0.512217    0.009449
   O    2.118672   -1.182126   -0.102757

4) 4.26 eV 5AC_CI3
   C    0.555701    1.517650   -0.113429
   C   -1.180383   -0.155212    0.095197
   C    1.066794   -0.571348    0.447798
   N   -0.740001    1.091624   -0.345015
```

```
   N   -0.224346   -0.613924    1.009291
   N    1.258741   -1.332840   -0.717429
   O   -2.227907   -0.628895   -0.181295
   H    1.196928   -2.317735   -0.561953
   H    2.110635   -1.102738   -1.185248
   H   -1.374155    1.644870   -0.876744
   H    0.742926    2.551985   -0.344942
   N    1.490498    0.784887    0.316275
```

Cyt:

```
1) 3.33 eV r_Cyt.run0032
   H   -2.286462    1.130118    0.038753
   N   -1.337910    0.852666   -0.084633
   C   -0.300662    1.732713    0.248881
   H   -0.321951    2.205008    1.220283
   C    0.970425    1.213234   -0.290253
   H    1.292383    1.538458   -1.267486
   C    1.147414   -0.232351   -0.004178
   N    2.395330   -0.719688    0.056694
   H    3.180874   -0.115729    0.096134
   H    2.515636   -1.687930    0.251126
   N    0.144297   -1.014901    0.127024
   C   -1.152470   -0.526046   -0.020113
   O   -2.097593   -1.252721   -0.079803

2) 3.45 eV s_Cyt.run0022
   H   -2.290315    1.103502   -0.033547
   N   -1.333955    0.831547    0.026012
   C   -0.300321    1.669254   -0.382072
   H   -0.522699    2.700249   -0.572388
   C    0.918654    1.213493    0.357926
   H    0.908060    1.376146    1.431315
   C    1.158887   -0.217375    0.015201
   N    2.417377   -0.661303   -0.071474
   H    3.185409   -0.034426   -0.051992
   H    2.570106   -1.616932   -0.302253
   N    0.172558   -1.018063   -0.145928
   C   -1.130667   -0.550739    0.005877
   O   -2.065217   -1.285201    0.110875

3) 3.53 eV r_Cyt2.run0094
   H   -2.283115    1.131434   -0.121879
   N   -1.342009    0.863934    0.067508
   C   -0.278309    1.694832   -0.309208
   H   -0.217359    2.026558   -1.335821
   C    0.923196    1.204734    0.379850
   H    0.975533    1.397404    1.441603
   C    1.157415   -0.220644    0.018639
   N    2.407592   -0.682574   -0.088414
   H    3.188020   -0.074031   -0.028732
   H    2.547596   -1.646137   -0.292466
   N    0.155603   -1.004347   -0.127066
   C   -1.146461   -0.521763    0.021629
   O   -2.086753   -1.252159    0.088455

 4) 3.82 eV r_Cyt.run0036a
   H    1.913986    1.328605    0.695069
```

N	1.132965	0.969125	0.194599
C	-0.048712	1.708327	0.105935
H	0.057938	2.762837	0.275444
C	-1.208495	1.125850	-0.166976
H	-2.117384	1.693513	-0.228361
C	-1.208770	-0.334511	-0.399830
N	-1.752486	-1.171918	0.588684
H	-2.552978	-0.784215	1.040433
H	-1.941100	-2.099167	0.267255
N	0.070411	-0.770378	-0.887477
C	1.160321	-0.374259	-0.082350
O	2.039657	-1.105227	0.242356

XYZ coordinates of CI structures

Part 2 Optimized with the OM2/MNDO approximation

sT:

1) 4.98 eV sT.run0010

C	1.3348123728	0.1806174589	0.0197134439
H	2.3860080650	0.1958369687	0.3094049064
N	0.5340671572	1.1948438280	0.0894284395
C	-0.8577981574	0.9441778752	-0.1409830852
H	-1.4623210721	1.8305471540	-0.3719790065
N	-1.4129814440	-0.2363066348	-0.0051717762
C	-0.3805874496	-1.1775402287	0.2234042609
H	-0.1947734403	-1.4055655751	1.2833374833
N	0.6794616365	-0.9210474590	-0.6262564413

2) 5.24 eV sT.run0054

C	1.7255776381	0.8138745168	0.4254535676
H	1.8579952700	1.8463814371	0.0996967347
N	1.3962713489	-0.2527650598	-0.0940480443
C	0.4679691501	-1.2364500623	-0.2765166037
H	0.8174921908	-2.2205434375	-0.6416555752
N	-0.7869424344	-1.0302727828	0.0554178186
C	-1.1801994581	0.2036056650	0.5642545384
H	-2.2669065856	0.2429085439	0.8175220609
N	-0.4383860653	1.2151511020	0.7232198262

AT:

1) 4.64 eV AT.run0033

C	1.3750007427	-1.2156568361	0.1396329657
H	2.1480231793	-2.0220576753	0.1494769619
N	0.1357817328	-1.4761118431	0.1898079568
C	-1.0565169779	0.0774779694	-0.4842076665
N	-2.1703990253	-0.4080732200	0.0300402636
H	-2.8150411240	-0.8736547294	-0.5811322546
H	-2.2319718508	-0.6643538491	1.0010153338
N	-0.2441648192	1.0196462588	-0.1676205945
C	1.0934695590	1.0923906426	0.0020172031
H	1.5294946066	2.1162749946	0.0062808203
N	1.9055482537	0.0582126237	0.0871560386

2) 4.73 eV AT.run0026

C	-0.7707156096	1.2137204679	-0.0875651377
H	-0.9721403991	2.2270945551	-0.4458395525
N	0.0871919751	0.9103911880	0.9714364436
C	0.9858172918	-0.0962592503	0.5790434642
N	1.6689627933	0.1891090196	-0.6153044697
H	2.2772541420	0.9835990368	-0.5482683977
H	2.1453325944	-0.5968524087	-1.0135518646
N	0.2292559199	-1.3307988990	0.3934204930
C	-0.9623860227	-1.0986264124	-0.0780897784
H	-1.6859722210	-1.9212108597	-0.2073546911
N	-1.4276471199	0.1693907525	-0.5107095438

3) 4.88 eV AT.run0003a

C	1.2569845134	0.9932547736	-0.2874169897

H	1.8249089141	1.8955232985	-0.5588116678
N	-0.1428178585	1.1592884755	-0.2215209191
C	-0.8533680630	0.0444329154	-0.1648826801
N	-2.1313501540	-0.0165402140	0.2590115932
H	-2.6985576970	-0.8045552938	0.0284625762
H	-2.6056374231	0.8262528813	0.4990957483
N	-0.0903825430	-1.1275496389	-0.5936856353
C	0.9455511097	-1.1181856853	0.3344681384
H	0.6622831198	-1.1764222930	1.3956475321
N	1.8882695200	-0.1070044272	0.0355171849

DT:

1) 4.63 eV DT2.run0025

C	-0.5298405382	1.6159066609	-0.0768927960
H	-0.6506551932	2.7038655063	-0.2381089961
N	0.7523883558	1.1265007352	-0.3463163278
C	0.9911914047	-0.1144126425	0.0763318775
N	2.0847374753	-0.8095829779	-0.2838258209
H	2.2915379641	-1.6949559410	0.1192891942
H	2.7356277952	-0.4228168387	-0.9293770280
N	0.0691118396	-0.5771598539	1.0659193694
C	-1.2389895818	-0.4941047661	0.5421484646
N	-1.3459470747	-1.2177063683	-0.6678419353
H	-1.3875873346	-2.2074635327	-0.5052952442
H	-2.1263494163	-0.9319545809	-1.2263652309
N	-1.5537682819	0.9029240177	0.2929014914

2) 4.71 eV DT.run0020

C	-0.0822938127	1.6042589370	-0.2161629095
H	-0.5123423229	1.6717687191	-1.2157507059
N	-0.9151641567	1.0112098819	0.7372516976
C	-1.1432905121	-0.3163106492	0.0971462979
N	-2.3661993884	-0.6795543497	-0.3478915755
H	-2.4705803053	-1.5318678647	-0.8518397322
H	-3.1835982832	-0.3245462243	0.1001466491
N	-0.0362538469	-0.9619408670	-0.2016585580
C	1.1659283060	-0.2000024510	-0.0241856387
N	2.2865819138	-0.9344902485	0.2306980676
H	2.2379930088	-1.9280082254	0.1635055565
H	3.1765746123	-0.5160082076	0.0563707225
N	1.2301882450	1.1305924138	-0.1328040691

3) 4.83 eV DT.run0092

C	-0.1997729120	1.6417751059	0.0107423257
H	-0.0967779504	2.7107545802	0.1799105042
N	0.8448485452	0.9579965660	-0.4858046636
C	1.3243800215	-0.1379413068	0.4199686975
N	2.4980806454	-0.7374276364	0.1163052045
H	3.3044864543	-0.5209770534	0.6507676241
H	2.5348843967	-1.5897643089	-0.4019201698
N	0.1544358526	-0.8577211982	-0.1064816187
C	-1.0685551331	-0.3683748193	0.0700314188
N	-2.1687587957	-1.1556583451	0.0869838907
H	-2.0959364549	-2.0850086479	-0.2462592147
H	-3.0442439579	-0.7063513123	-0.0459553665
N	-1.3307593024	1.0256038720	0.2533089486

TT:

1) 4.89 eV TT2.run0011

C	1.3732670733	-0.4861910229	-0.5821660859
N	2.4605346144	-1.0055644385	-0.0120187037
H	2.7386398356	-1.9268887577	-0.2762967708
H	2.7831880898	-0.7072913700	0.8915944503
N	0.0420070575	-1.3338919617	0.5623093101
C	-1.1152399632	-0.8077808991	0.2455745846
N	-2.2151233784	-1.5564139818	-0.0542877191
H	-2.1995068664	-2.5462107947	0.0410239207
H	-3.0921605977	-1.1201896726	-0.2244540407
N	-1.2911681237	0.5625003469	0.2170641006
C	-0.1943262916	1.2708505767	-0.0970759127
N	-0.2962183438	2.6322813755	-0.1116036209
H	-1.1811548881	3.0580764432	0.0434438935
H	0.4208111193	3.1827088440	-0.5284573134
N	1.0120439135	0.7792534710	-0.5068263928

2) 5.37 eV TT2.run0036

C	-1.2143914662	0.7018768080	-0.4137388965
N	-2.4071244242	1.2839285206	-0.1601088082
H	-2.8591113579	1.2522542683	0.7255743589
H	-2.8513857401	1.8109940666	-0.8684515784
N	-0.0890481444	1.1699787230	0.4171051951
C	1.1101245129	0.6435284611	0.0408053414
N	2.1396093283	1.5067787470	-0.1760596325
H	1.9973983305	2.4658390365	0.0415826051
H	3.0401904696	1.1529228028	-0.2614975084
N	1.3380178571	-0.6964985161	-0.1121079368
C	0.0868153954	-1.2890383281	-0.0236767195
N	0.0237534832	-2.6332227942	-0.0870444761
H	0.8501819299	-3.1600423306	-0.1065202958
H	-0.8525666460	-3.0844755535	-0.0443086321
N	-1.0294106753	-0.5886880646	0.2651337178

5AC:

1) 4.15 eV 5AC.run0023

H	-1.6441553685	1.7994413923	-0.6485074744
N	-0.9572361417	1.2177245295	-0.1876621314
C	0.3552386508	1.6676077730	-0.0089365607
H	0.5204176093	2.7629389970	-0.0724131171
N	1.3283966034	0.8609396909	0.2674828637
C	1.0378132763	-0.5609106579	0.3708301550
N	1.1482718470	-1.1792719080	-0.8848892715
H	0.9716809376	-2.1710430234	-0.8834760070
H	1.9976063603	-0.9551167857	-1.3745215387
N	-0.2584299978	-0.6659225347	0.9267796185
C	-1.2864655567	-0.1257950262	0.0607299506
O	-2.2973811387	-0.6889919193	-0.3431915017

2) 4.24 eV 5AC.run0063a

H	2.1624463279	1.1892140119	-0.0376677201
N	1.3046172549	0.7475931391	0.2597467609
C	0.0996102551	1.4758606274	0.2681029967
H	-0.2298171281	1.8753406379	1.2561235652
N	-0.8697095690	0.6671814966	-0.3995910785

C	-1.0611075591	-0.4126393288	0.5598045911
N	-2.3400194416	-0.7682386408	0.8327395050
H	-2.4997333827	-1.6075998707	1.3524309623
H	-3.1054733955	-0.3932063486	0.3149342944
N	-0.0539986897	-1.0226011322	1.1397147875
C	1.2654049860	-0.5210944214	0.8283424962
O	2.2849657620	-1.1809751224	1.0290795290

3)4.28 eV 5AC.run0013

H	2.0595567737	1.4518961741	-0.2554424169
N	1.2547192105	0.8805029767	-0.0692062874
C	0.0670321208	1.5985763016	0.2569780816
H	-0.2142769654	1.5797866186	1.3186765528
N	-0.9379730053	1.0687741721	-0.6400033043
C	-1.1130760567	-0.2844886310	-0.0868074706
N	-2.4005985095	-0.6993002518	0.1537092622
H	-2.5457958768	-1.6842689097	0.3222375469
H	-3.1496871430	-0.1452989798	-0.1644952378
N	-0.0775755990	-1.0695734932	0.1492330857
C	1.2176182995	-0.4828507552	-0.0661432068
O	2.2320640321	-1.1774399171	-0.2399295007

Cyt:

1) 3.49 eV Cyt.run0021a

H	2.2987839022	1.1988062521	-0.1275786807
N	1.3506413397	0.9121975059	0.1132818178
C	0.2930476966	1.6169669182	-0.3492302604
H	0.3070143010	2.2294750947	-1.2442722804
C	-0.9259424054	1.2501504218	0.3132793577
H	-0.9955509842	1.4774120834	1.3995995128
C	-1.1782964322	-0.2017128722	0.0487778618
N	-2.4240461095	-0.6576054501	-0.1826804611
H	-3.2034421447	-0.0384219248	-0.2300384657
H	-2.5911846589	-1.6316305791	-0.3210601194
N	-0.1534247845	-1.0343586140	0.1235241637
C	1.1290845314	-0.5262954605	0.3132130968
O	2.1291566505	-1.2002411235	0.5745093188

2) 3.74 eV Cyt.run0011

H	1.6266673974	1.5260657757	-0.9531769952
N	0.9243645677	1.1023156420	-0.3645982127
C	-0.3433606240	1.6788288910	-0.2566582290
H	-0.4395671336	2.7438251895	-0.4883372891
C	-1.3905256100	0.9122435200	0.1536695444
H	-2.3975918535	1.2956434847	0.2588309329
C	-1.1915582033	-0.5315795609	0.5033928196
N	-1.3599227945	-1.3867262269	-0.5930696321
H	-2.1495790601	-1.1766944443	-1.1775041514
H	-1.3288705038	-2.3630369079	-0.3552057054
N	0.1120898233	-0.5735122492	1.0538968417
C	1.1509708311	-0.1767216817	0.1236432365
O	2.1312743711	-0.8617448691	-0.1724846042
